# A van der Waals Model of Solvation Thermodynamics

**DOI:** 10.3390/e26080714

**Published:** 2024-08-22

**Authors:** Attila Tortorella, Giuseppe Graziano

**Affiliations:** 1 Scuola Superiore Meridionale, Via Mezzocannone, 4, 80138 Naples, Italy; attila.tortorella@unina.it; 2 Department of Chemical Sciences, University of Naples Federico II, Via Cintia, 4, 80126 Naples, Italy; 3 Department of Science and Technology, University of Sannio, Via Francesco de Sanctis, SNC, 82100 Benevento, Italy

**Keywords:** van der Waals model of liquids, solvation, number density, molecular size, repulsive and attractive interactions

## Abstract

Exploiting the van der Waals model of liquids, it is possible to derive analytical formulas for the thermodynamic functions governing solvation, the transfer of a solute molecule from a fixed position in the ideal gas phase to a fixed position in the liquid phase. The solvation Gibbs free energy change consists of two contributions: (a) the high number density of all liquids and the repulsive interactions due to the basic fact that each molecule has its own body leading to the need to spend free energy to produce an appropriate cavity to contain the solute molecule; (b) the ubiquitous intermolecular attractive interactions lead to a gain in free energy for switching-on attractions between the solute molecule and neighboring liquid molecules. Also the solvation entropy change consists of two contributions: (a) there is an entropy loss in all liquids because the cavity presence limits the space accessible to liquid molecules during their continuous translations; (b) there is an entropy gain in all liquids, at room temperature, due to the liquid structural reorganization as a response to the perturbation represented by solute addition. The latter entropy contribution is balanced by a corresponding enthalpy term. The scenario that emerged from the van der Waals model is in qualitative agreement with experimental results.

## 1. Introduction

Water and aqueous solutions play a fundamental role on the Earth and for living organisms. All biochemical processes occur in aqueous solutions, taking advantage of the extraordinary properties of water [1,2]. For instance, the ability of polypeptide chains to fold and assume a unique 3D structure, the native, functionally active one, is strictly connected to the gain in translational entropy of water molecules for the increase in accessible configurational space associated with chain collapse [3]. The quantitative study, by both experimental approaches and theoretical methods, of phenomena related to the hydrophobic effect pointed out the need for a profound change in the way to analyze data in order to arrive at correctly defined thermodynamic quantities [1]. Most of the needed work has been performed by Arieh Ben-Naim, as it can readily be recognized on looking at the three books he published in 1974, 1980 and 1987 [4,5,6]. Following Ben-Naim’s statistical mechanical analysis, solvation describes the process of transferring a solute molecule from a fixed position in the ideal gas phase to a fixed position in the liquid phase (the latter can be a pure liquid or a solution, without restrictions on the concentration of the components). The transfer of a species from a liquid to another liquid is defined in the same way. This definition allows the elimination of the effects coming from the difference in molar volume of the two phases, that can be large [7,8] (i.e., for the transfer from the ideal gas phase to water, the Gibbs free energy contribution coming from the simple difference in molar volume amounts to 17.87 kJ mol^−1^, at 25 °C and 1 atm; for the transfer from n-hexane to water, it amounts to 4.92 kJ mol^−1^ at 25 °C and 1 atm). Ben-Naim showed that the effects due to the difference in molar volume and thermal motion can be eliminated not only in theoretical approaches, but also in analyzing experimental data by simply using the molar concentration scale [5,6].

Now the use of the so-called Ben-Naim standard is almost universally accepted, but, at the beginning, several scientists raised a lot of concerns. In particular, in the nineties, it was erroneously asserted that a correction term, accounting for the difference in molar volume between solute and solvent, should be added to the transfer Gibbs free energy values based on the molar concentration scale [9,10,11,12]. In retrospect, these erroneous claims were motivated by a wrong understanding of the deep analysis performed by Ben-Naim [13,14,15,16].

In the present study, we want to use the van der Waals model of real gases and liquids to develop a statistical mechanical approach to solvation thermodynamics. In 1873, in his PhD thesis, van der Waals proposed the famous and successful equation of state, named after him [17]. He was able to account in a very simple manner for: (1) the volume possessed by each molecule that leads to the non-accessibility of the space occupied by the other molecules, as a consequence of strongly repulsive and short-range intermolecular interactions; (2) the long-range intermolecular energetic attractions. These two corrections take the form of the two van der Waals constants: the repulsive one, *b*, and the attractive one, *a*, which are specific for each chemical species. The van der Waals equation of state, even though very simple, is able to describe the gas–liquid phase transition, to reproduce the existence of the critical point and of the law of corresponding states, and to lead to a virial series expansion [18,19]. The van der Waals model is correct from a qualitative point of view, not from the quantitative one. It may be useful to describe solvation thermodynamics because the treatment is entirely analytic [20,21,22], and the various formulas have a transparent physical meaning.

## 2. A van der Waals Binary Solution

In this study, we consider monoatomic particles to eliminate the subtleties associated with the presence of internal degrees of freedom. Starting from the physico-chemical ideas of van der Waals for a binary solution of monoatomic species, it is possible to write down the corresponding classical partition function [20,21]:

(1) $$\begin{array}{cc} Q\left(N_{1},N_{2},V,T\right) & =\left[\frac{V^{\left(N_{1}+N_{2}\right)}}{N_{1}!{\Lambda_{1}}^{3N_{1}}\cdot N_{2}!{\Lambda_{2}}^{3N_{2}}}\right]\cdot {\left[\frac{V-\left(b_{1}N_{1}+b_{2}N_{2}\right)}{V}\right]}^{\left(N_{1}+N_{2}\right)}\\ & \cdot \exp\left[\frac{{\left(a_{1}N_{1}+a_{2}N_{2}\right)}^{2}}{kTV}\right] \end{array}$$

where *V* is the volume of the system; Λ_i_ ≡ *h*/(2πm_i_*kT*)^1/2^ is the thermal de Broglie wavelength of particles i; *N*_i_ is the number of particles i; the parameter *b*_i_ measures the repulsive interactions of particles i, and the parameter *a*_i_ measures the attractive interactions of particles i (i.e., *a*_i_ and *b*_i_ are positive and constant, because they are considered to be independent of temperature); *k* is the Boltzmann constant and *T* is the absolute temperature. The quantity [*V* − (*b*_1_*N*_1_ + *b*_2_*N*_2_)] represents the free volume of the system, and, according to van der Waals, the particles move independently of one another in this volume, where there is a constant attractive potential energy given by −(*a*_1_*N*_1_ + *a*_2_*N*_2_)^2^/*V* (i.e., the absence of a potential energy gradient implies the absence of attractive intermolecular forces). Knowledge of the canonical partition function allows the straightforward calculation of the Helmholtz free energy, and then of the chemical potential of component 2 in the solution:

(2) $$\mu_{2}\;\equiv \;{\left(\frac{\partial A}{\partial N_{2}}\right)}_{T,V,N_{1}}\;$$

Performing the calculations, one arrives at [21]:

(3) $$\begin{array}{cc} \mu_{2}\left(vdW\right)= & kT\cdot ln\rho_{2}{\Lambda_{2}}^{3}-kT\cdot \ln\left[\frac{V-(b_{1}N_{1}+b_{2}N_{2})}{V}\right]+kT\cdot \left[\frac{\left(N_{1}+N_{2}\right)b_{2}}{V-\left(b_{1}N_{1}+b_{2}N_{2}\right)}\right]\\ & -\frac{2a_{2}\left(a_{1}N_{1}+a_{2}N_{2}\right)}{V} \end{array}$$

where ρ_2_ = *N*_2_/*V* is the number density of component 2. Equation (3) represents the chemical potential of component 2 in a binary solution of monoatomic van der Waals fluids. The expression indicates that the classical translational degrees of freedom are separated from the terms accounting for all the interactions of a component 2 particle with the surroundings. Therefore, the chemical potential can be rewritten as:

(4) $$\mu_{2}\left(vdW\right)=kT\cdot \ln\rho_{2}{\Lambda_{2}}^{3}+{\mu_{2}}^{•}$$

where *μ*_2_• consists of the three remaining terms in Equation (3) and represents the standard chemical potential according to Ben-Naim (we denote the Ben-Naim standard quantities by means of a superscript filled circle); it represents the coupling work of the solute molecule to the solution, or the interaction free energy of a component 2 particle, fixed at any position in the solution, with its surroundings, without accounting for special limits to the concentration of the solute. This physical interpretation is a direct consequence of the adopted statistical mechanical procedure. It is not necessary to define a special state, the so-called standard state, in contrast to what happens in the framework of classical thermodynamics. The quantity *μ*_2_• is affected by the solution composition, precisely because the coupling work magnitude depends on the solution composition [6,7,8]. This is a fundamental feature of the so-called Ben-Naim standard chemical potential, and its advantage in order to address the role of solvation thermodynamics in basic biochemical processes, such as protein folding, protein–protein and protein–DNA recognition because the latter occur in crowded media [23].

As it is well known, the expression of the chemical potential of the monoatomic component 2 in the ideal gas phase is [19]:

(5) $$\mu_{2}\left(ig\right)=kT\cdot ln\rho_{2}{\Lambda_{2}}^{3}$$

There are no interactions among ideal gas molecules, and so only the classical translational degrees of freedom contribute to *μ*_2_(ig), and *μ*_2_• = 0. Statistical thermodynamics unequivocally indicates that the number density is the concentration unit to be used in the chemical potential formulas.

## 3. Solvation Thermodynamics

The knowledge of the chemical potential of the component 2 in the van der Waals binary solution and in the ideal gas phase allows us to study solvation thermodynamics, assuming that the liquid state can be described as a van der Waals binary solution. At thermodynamic equilibrium, the chemical potential of the component 2 has to be the same in the two phases:

(6) $$\mu_{2}\left(ig\right)=\mu_{2}\left(vdW\right)$$

Inserting Equations (4) and (5) into Equation (6) leads to:

(7) $$kT\cdot ln\rho_{2}\left(ig\right){\Lambda_{2}}^{3}={\mu_{2}}^{•}\left(vdW\right)+kT\cdot ln\rho_{2}\left(vdW\right){\Lambda_{2}}^{3}$$

The thermal de Broglie wavelength term is identical in the two phases and cancels out, and one has:

(8) $$\Delta G^{•}={\mu_{2}}^{•}\left(vdW\right)=kT\cdot \ln\left[\frac{\rho_{2}\left(ig\right)}{\rho_{2}\left(vdW\right)}\right]$$

where Δ*G*^•^ is the Ben-Naim standard Gibbs free energy change associated with the solvation process. There are two equalities in Equation (8); the first indicates that Δ*G*^•^ is given by the solute–solvent coupling work; the second indicates that Δ*G*^•^ can be calculated by determining the ratio of the number densities of component 2 in the two phases, at constant temperature and pressure (i.e., Δ*G*^•^ values are readily obtained from experimental data) [6,7].

Now, we consider a solution of the component 2 in the monoatomic van der Waals fluid 1, at infinite dilution. The Δ*G*^•^ expression is readily obtained by making the limit in Equation (3), noting that for *N*_2_ → 0, *N* ≅ *N*_1_ and *V* ≅ *N*_1_*v*_1_, where *v*_1_ is the molecular volume of component 1. Thus, Δ*G*^•^ is given by [21]:

(9) $$\Delta G^{•}={\mu_{2}}^{•}\left(vdW\right)=-kT\cdot \ln\left[\frac{v_{1}-b_{1}}{v_{1}}\right]+kT\cdot \left[\frac{b_{2}}{v_{1}-b_{1}}\right]-2\left(\frac{a_{1}a_{2}}{v_{1}}\right)$$

To proceed with our analysis, it is helpful to assign a physical meaning to the contributions making up Equation (9). The first two terms correspond to the reversible work to produce an appropriate cavity to contain the component 2 particle in the monoatomic van der Waals fluid 1:

(10) $$\Delta G_{c}\left(vdW\right)=-kT\cdot \ln\left[\frac{v_{1}-b_{1}}{v_{1}}\right]+kT\cdot \left[\frac{b_{2}}{v_{1}-b_{1}}\right]$$

For the first term of Equation (10), we can write:

(11) $$-kT\cdot \ln\left[\frac{v_{1}-b_{1}}{v_{1}}\right]=-kT\cdot \ln\left[\frac{v_{1,\;free}}{v_{1}}\right]=-kT\cdot \ln\left(1-\xi_{1}\right)$$

and for the second term:

(12) $$kT\cdot \left[\frac{b_{2}}{v_{1}-b_{1}}\right]≅\;P\left(vdW\right)\cdot b_{2}\;$$

where Equation (11) represents the decrease in the configurational space that occurs when inserting the component 2 particle (note that ξ_1_ is the volume packing density of fluid 1), and Equation (12) represents the pressure–volume work done to insert the component 2 particle in the van der Waals fluid 1. The two terms in Equation (10) should correspond to the first and fourth terms in the Δ*G*_c_ formula provided by the classic scaled particle theory [24,25,26]. This theory uses geometric criteria to take into account the spatial correlations existing between the particles in any liquid phase for the simple fact that each particle has its own body and so two particles cannot stay in the same position, at the same instant of time (i.e., non-overlap requirement). Scaled particle theory gives quantitative predictions that are more accurate than those from Equation (10) [22,27].

The physical interpretation of the parameters *a*_1_ and *a*_2_ leads to state that the third term on the right-hand-side of Equation (9) is the attraction Gibbs free energy of the component 2 particle with all the particles of the van der Waals fluid 1:

(13) $$\Delta G_{a}\left(vdW\right)=-\frac{2a_{1}a_{2}}{v_{1}}$$

Equation (9) shows that the solvation process can be decomposed in two sub-processes [4,5,6,7,24,25,26,27,28,29]: (a) production of an appropriate cavity in the liquid to contain the solute molecule, Equations (10)–(12); (b) switching-on the attractive potential energy between the solute molecule and the neighboring solvent molecules, Equation (13). Solvation thermodynamics is ruled by the effect of the solvent-excluded volume associated with cavity production in the liquid, and by the solute–solvent energetic attractions [4,5,6,7]; the quantity Δ*G*^•^ accounts for both such contributions, as obtained in the van der Waals model [20,21].

It is straightforward to calculate the enthalpy and entropy changes associated with the two sub-processes under the constant pressure condition [21]. For cavity production, the following relationships are obtained:

(14) $$\Delta H_{c}=-T^{2}\left[\frac{\partial \left(\frac{\Delta G_{c}}{T}\right)}{\partial T}\right]=kT^{2}\left[\frac{\alpha_{P,1}}{v_{1}-b_{1}}\right]\cdot \left\{b_{1}+\left[\frac{v_{1}b_{2}}{v_{1}-b_{1}}\right]\right\}$$

(15) $$\begin{array}{cc} \Delta S_{c}=-\left(\frac{\partial \Delta G_{c}}{\partial T}\right) & =k\cdot \ln\left[\frac{v_{1}-b_{1}}{v_{1}}\right]-k\cdot \left[\frac{b_{2}}{v_{1}-b_{1}}\right]+\left(\frac{\Delta H_{c}}{T}\right)\\ & =\Delta S_{x}+\left(\frac{\Delta H_{c}}{T}\right) \end{array}$$

(16) $$T\cdot \Delta S_{c}=T\cdot \Delta S_{x}+\Delta H_{c}\;$$

where Δ*S*_x_ = −Δ*G*_c_/*T*, and represents the solvent-excluded volume entropy contribution due to cavity production; and α_P,1_ is the isobaric thermal expansion coefficient of the van der Waals fluid 1. A liquid structural reorganization occurs upon cavity production; this contribution is directly proportional to α_P,1_. We see from Equation (14) that Δ*H*_c_ is proportional to α_P,1_ and from Equation (16) that *T*·Δ*S*_c_ increases with Δ*H*_c_, hence any increase in α_P,1_ yields the same increment in both Δ*H*_c_ and *T*·Δ*S*_c_, and thus no change in Δ*G*_c_. We can conclude that, in terms of liquids with different α_P,1_, cavity production yields compensating enthalpy and entropy changes. The production of a cavity, under *NPT* conditions, causes a small change in the volume of the system, which, in turn, determines an enthalpy change (remember that the internal energy of a van der Waals fluid is inversely proportional to the volume). This enthalpy change has to come from the liquid structural reorganization since there is no other source, and is totally balanced by a corresponding entropy change. For switching-on the attractive potential, the following relationships are obtained:

(17) $$\Delta H_{a}=-T^{2}\left[\frac{\partial (\frac{\Delta G_{a}}{T})}{\partial T}\right]=-\left(\frac{2a_{1}a_{2}}{v_{1}}\right)\cdot \left(1+\alpha_{P,1}T\right)$$

(18) $$\Delta S_{a}=-\left(\frac{\partial \Delta G_{a}}{\partial T}\right)=\frac{-2a_{1}a_{2}\cdot \alpha_{P,1}}{v_{1}}$$

(19) $$T\cdot \Delta S_{a}=\left(\frac{-2a_{1}a_{2}}{v_{1}}\right)\cdot T\cdot \alpha_{P,1}\;$$

(20) $$\Delta H_{a}=\left(\frac{-2a_{1}a_{2}}{v_{1}}\right)+T\cdot \Delta S_{a}$$

where again the liquid structural reorganization upon switching-on the solute–solvent attractive potential depends linearly on α_P,1_, and affects the enthalpy and entropy changes in a totally compensating manner. The finding that the liquid structural reorganization depends linearly on the isobaric thermal expansion coefficient of the liquid cannot be a surprise because α_P_ comes from the ensemble correlations between enthalpy fluctuations and volume fluctuations: α_P_ ≡ <δ*H*·δ*V*>/*kT*^2^<*V*>, where δ*H* = *H* − <*H*> and δ*V* = *V* − <*V*> represent the enthalpy and volume fluctuations with respect to their ensemble average values [19].

Such results are in line with the analysis performed by Qian and Hopfield with the aim to rationalize the presence, in several and different processes, of a large enthalpy-entropy compensation [30]. It was shown that the thermodynamic effect of a small perturbation on a system consists of two contributions: (1) one accounting for the direct interaction of the perturbation with the untouched system; (2) the other accounting for the system’s response to the perturbation, by realizing a redistribution among its different microstates, which are in thermal equilibrium, in accord with the Le Chatelier principle. The external constraints imposed to the system determine the allowed microscopic fluctuations, and govern the redistribution. The latter proves to be characterized by a complete enthalpy-entropy compensation, and the Gibbs free energy change comes from the direct interaction between the perturbation and the untouched system. The perturbation, in the case of the solvation process, is given by the addition of the solute molecule to a fixed position of the solvent. The direct part of the perturbation consists of both cavity production and switching-on solute–solvent attractive potential; the structural reorganization of the liquid is the response of the system to the perturbation.

## 4. Some Calculations

It may be instructive to make some calculations with the van der Waals model of solvation thermodynamics. In particular, we calculate the reversible work to produce an appropriate cavity to contain a xenon atom (i.e., its hard sphere diameter σ = 4 Å) in water, methanol, ethanol, carbon tetrachloride, n-hexane, n-decane, c-hexane and benzene, at 25 °C and 1 atm. This choice is dictated by the understanding that it is the cavity production step that distinguishes water from the other common liquids [4,7,25,27]. To perform calculations, it is necessary to modify Equation (10), converting to molar quantities and rearranging the terms [22]:

(21) $$\Delta G_{c}\left(vdW\right)=-RT\cdot \ln\left(1-\rho_{1}b_{1}\right)+RT\cdot \left[\frac{\rho_{1}b_{2}}{1-\rho_{1}b_{1}}\right]$$

where ρ_1_ = *N*_Av_/*v*_1_ is the liquid number density, *v*_1_ is its molar volume, *b*_i_ = (π·σ_i_^3^/6)/0.64, σ_i_ is the effective hard sphere diameter of liquid molecules or of the solute particle, and the factor 0.64 in the denominator accounts for the basic fact that the random close packing of spheres corresponds to 64% of system volume occupancy [31]. The cavity enthalpy and entropy changes, expressed as Δ*G*_c_(vdW) in Equation (21) in terms of molar quantities, are:

(22) $$\Delta H_{c}\left(vdW\right)=RT^{2}\cdot \alpha_{P,1}\cdot \left[\frac{\rho_{1}}{1-\rho_{1}b_{1}}\right]\cdot \left\{b_{1}+\left[\frac{b_{2}}{1-\rho_{1}b_{1}}\right]\right\}$$

(23) $$\Delta S_{c}\left(vdW\right)=R\cdot \ln\left(1-\rho_{1}b_{1}\right)-R\cdot \left[\frac{\rho_{1}b_{2}}{1-\rho_{1}b_{1}}\right]+\frac{\Delta H_{c}\left(vdW\right)}{T}$$

To perform calculations, we have used the experimental values of *v*_1_ and α_P,1_ for the eight liquids, and their effective hard sphere molecular diameters [16,32] (see the columns 2–4 of Table 1). It is important to recognize that it is a very rough approximation to consider as simple spheres the molecules of n-hexane or n-decane, but also those of ethanol and benzene. The calculated values are reported in the columns 6–10 of Table 1. It results that: (a) the Δ*G*_c_(vdW) value in water is significantly larger than in the other liquids; (b) the Δ*H*_c_(vdW) value in water is significantly smaller than in the other liquids; in addition, Δ*H*_c_(vdW) is markedly smaller than Δ*G*_c_(vdW) in water, but the reverse holds in the other liquids, with the exception of methanol; (c) the Δ*S*_c_(vdW) value is large and negative in water, but small and positive in the other liquids, again with the exception of methanol.

Since the calculations have been performed in the same way, regardless of the liquid identity, the differences have to come from the physico-chemical properties of the considered liquids. The Δ*G*_c_(vdW) magnitude depends on the liquid number density, and water has the largest ρ_1_ value because it has the smallest molar volume, which reflects the very small diameter of water molecules [32]. The Δ*S*_c_(vdW) formula consists of two contributions: (a) the first Δ*S*_x_ = −(Δ*G*_c_/*T*) is a measure of the solvent-excluded volume effect associated with cavity creation, and is always negative in all liquids; (b) the second Δ*S*_nx_ = (Δ*H*_c_/*T*) is a measure of the liquid structural reorganization upon cavity creation, is proportional to α_P,1_, and is always positive at room temperature in all liquids. The contribution of the liquid structural reorganization is larger in the organic liquids than in water due to the α_P,1_ magnitude (i.e., the latter depends on the strength of the intermolecular attractions in the liquid, 3D H-bonds in water versus dispersion attractions in hydrocarbons); in addition, it is able to overwhelm the Δ*S*_x_ contribution in the organic liquids, rendering Δ*S*_c_(vdW) positive. In contrast, the contribution of the liquid structural reorganization is not large in water, and cannot counterbalance the large and negative Δ*S*_x_ contribution, so that Δ*S*_c_(vdW) is negative. It is worth underscoring that the Δ*G*_c_(vdW) values in all the considered liquids are markedly smaller than those calculated by means of scaled particle theory or computer simulations, because the van der Waals model accounts in a very approximate manner for the solvent-excluded volume effect [22,27,32]. This reflects in the Δ*S*_x_ = −(Δ*G*_c_/*T*) magnitude, that proves to be too small in all the considered liquids [32,33]. As a consequence, one cannot expect that the van der Waals model can reproduce in a quantitative manner the solvation thermodynamics of xenon or other solutes in the considered liquids. Its merits consist in the possibility to arrive at analytical, and qualitatively correct relationships for all the relevant thermodynamics quantities.

## 5. Conclusions

The present analysis and calculations emphasize that the van der Waals model of solvation works in a qualitatively correct manner because it recognizes the need, in liquid phases, to account for the short-range repulsive interactions that produce the solvent-excluded volume effect on solute addition [34,35]. The latter, however, is treated in an approximate manner, because the van der Waals model accounts only for the liquid free volume (1 − ρ_1_*b*_1_). In real liquids, the free volume represents 50–60% of the total volume, whereas the appropriate volume to contain a molecular-sized solute is smaller by orders of magnitude, because the solvent-excluded volume effect is operative (i.e., the non-overlap requirement holds) [36]. Only a very small part of the liquid’s free volume is available to host a real solute (i.e., only the cavities large enough to contain the solute molecule). These sentences underscore that the free volume division is a fundamental feature of a liquid (largely dependent on the size and shape of the liquid molecules), that is totally neglected by the van der Waals model. In any case, the latter, using the experimental values of ρ_1_ and α_P,1_, leads to a clear difference between water and the organic liquids for the cavity entropy change, in line with scaled particle theory results [7,25,27].

## Figures and Tables

**Table 1:** Effective diameters, experimental values, at 25 °C and 1 atm, of the molar volume and isobaric thermal expansion coefficient, and *b*_1_ values for the eight considered liquids [32]. Gibbs free energy change, enthalpy and entropy change for the creation in the considered liquids of a cavity of 4 Å diameter, using the van der Waals model, Equations (21)–(23). The two contributions of the cavity entropy change are reported in the last two columns. For xenon, *b* = 52.4 Å^3^.

	σ_1_ Å	*v*_1_ cm^3^ mol^−1^	α_P,1_·10^3^ K^−1^	*b*_1_ Å^3^	Δ*G*_c_ kJ mol^−1^	Δ*H*_c_ kJ mol^−1^	Δ*S*_c_ J K^−1^mol^−1^	Δ*S*_x_ J K^−1^mol^−1^	Δ*H*_c_/*T* J K^−1^mol^−1^
water	2.80	18.07	0.257	18.0	13.0	2.3	−35.9	−43.6	7.7
methanol	3.83	40.73	1.189	46.0	8.8	8.5	−1.0	−29.5	28.5
ethanol	4.44	58.68	1.089	71.6	8.3	8.4	0.4	−27.8	28.2
CCl_4_	5.37	97.09	1.226	126.7	7.6	9.7	7.0	−25.5	32.5
n-hexane	5.92	131.62	1.390	169.7	6.5	8.8	7.7	−21.8	29.5
n-decane	7.08	195.94	1.020	290.3	9.2	16.5	24.4	−30.9	55.3
c-hexane	5.63	108.75	1.214	146.0	7.9	10.9	10.1	−26.5	36.6
benzene	5.26	89.40	1.240	119.1	8.5	12.0	11.7	−28.5	40.2

## Data Availability

All the relevant data are reported in the article.
